# Exploring end-of-life interaction in dyads of parents and adult children: a protocol for a mixed-methods study

**DOI:** 10.1186/s12904-018-0322-4

**Published:** 2018-04-27

**Authors:** Stephanie Stiel, Eva-Maria Stelzer, Nils Schneider, Franziska A. Herbst

**Affiliations:** 1 Institute for General Practice, Hannover Medical School, Carl-Neuberg-Straße 1, 30625 Hanover, Germany; 2 Department of Psychology, The University of Arizona, 1503 E University Blvd, Tucson, AZ 85721 USA

**Keywords:** Palliative care, End-of-life care, Terminal illness, Dyadic interaction, Interpersonal relations, Informal caregivers, Health services research, Grounded Theory, Psychology

## Abstract

**Background:**

A considerable number of terminally-ill adult children are outlived by at least one parent and receive palliative care prior to their death. At the same time, adult children continue to be confronted with their parents’ terminal illnesses and end-of-life situations. The current study explores the specifics of dyadic interaction at the end of life between a) adult children suffering from a life-threatening disease and their parents, and b) terminally ill parents and their adult children.

**Methods:**

This prospective observational study aims at filling the existing gap on adult child-parent interaction specifics at the end of life using an exploratory mixed-methods framework. The mixed-methods framework combines a qualitative face-to face interview and quantitative self-report questionnaires to study the topic at hand. The qualitative interview will focus on experiences, expectations, and wishes with regard to dyadic communication, information about illness and prognosis, expressed and perceived burden and support as well as caregiving role at the end of life. The questionnaires will cover socio-demographics, loneliness, attachment style, social support, and emotional closeness.

**Discussion:**

The research group is currently adjusting a semi-structured interview guide and questionnaire instructions based on the results of a multiprofessional scientific advisory board meeting (Jan. 2018). In a next step, and prior to qualitative and quantitative data collection, the questionnaires will be piloted on patients and their family members in a palliative care setting. The main expected results are i) a description of the specifics of the interaction within and between both dyads, ii) the development of hypotheses and a theoretical framework on the specifics, similarities, and differences for both study groups, and iii) clinical conclusions on specific psychosocial care needs of both groups.

**Trial registration:**

The study was registered prospectively in the Health Services Research Germany register (Versorgungsforschung Deutschland – Datenbank) (Registration N° VfD_Dy@EoL_17_003897; date of registration: November 22, 2017) and in the German Clinical Trials Register (Deutsches Register Klinischer Studien) (Registration N° DRKS00013206; date of registration: October 27, 2017). The study is visible in the International Clinical Trials Registry Platform Search Portal of the World Health Organization under the German Clinical Trials Register number.

## Background

With an aging society, the number of parents who are confronted with the death of their adult child is rising. In Germany, approximately 16,000 adults die every year before the age of 45 [1]. A considerable percentage of them is being survived by at least one parent and receives palliative care (PC) prior to their death. At the same time, adult children continue to be confronted with their parents’ terminal illnesses and end-of-life (E-o-L) situations. According to the World Health Organization definition, PC seeks to improve the quality of life of both patients and their families facing life-threatening illness [2]. In line with this principle, the current study will apply a dyadic framework to examine interaction specifics at the end of life (EoL) between a) adult children suffering from a life-threatening disease and their parents and b) terminally ill parents and their adult children.

### Investigating interaction in dyads of parents and adult children

The current study focuses on the subpopulation parents-adult children since this dyad may face specific challenges with regard to communication, information about illness, expressed and perceived burden and support as well as caregiving role during PC hospitalization. So far, there is a paucity of data on interactions between parents and adult children at the EoL even though personalized health care is becoming increasingly popular. Likewise, no evidence-based psychosocial interventions exist for this dyad. Retrospective case studies examining psychosocial and physical health outcomes and levels of disordered grief found parents to experience mental and physical health impairment following their child’s life-threatening diagnosis and subsequent death [3]. Examining the specifics of the interaction between adult children suffering from a life-threatening disease and their parents in comparison to the interaction of terminally ill parents and their children may provide better understanding of the distinct psychosocial needs of both dyads and may decrease the likelihood of developing negative health outcomes while increasing quality of life.

Being the relative of a terminally ill family member (FM) can become emotionally, physically, and socially stressful [4–10]. Amongst other aspects, caregivers of PC patients are challenged by fear of loss, mental health problems, and trouble sleeping [5, 6, 8, 10]. Studies assessing patients’ quality of life and burden on family caregivers generally reported interdependencies between the two. One study [11] found a relationship between burden perceived by family caregivers and patients’ feelings of anxiety and depression. Another study [10] showed increases in caregivers’ depression and perceived burden as a function of patients’ declining functional status. Additional challenges in interaction at the EoL may be faced by adult children who have been diagnosed with a life-limiting disease and by their surviving parents. Parents of terminally ill adult children experience not only a multiplicity of intense emotional feelings including uncertainty, helplessness, and injustice over the reversed natural order of death [12–16], but they also report being excluded from their child’s decision-making process [16]. This may result in uncertainty and loss of control with regard to the course of the disease, treatment effects, and time left with their sick child. Another burdensome factor is the lack of open communication about illness and fears between sick children and parents [14, 16]. The child’s life-limiting disease may further trigger the parental wish to provide care for their adult child and to be present at the time of death. This desire may be in conflict with their adult child’s sense of autonomy and the desire of their child’s romantic partner to serve as a main caretaker [12, 17]. Hence, parents describe a transformation of the parent role from an active and directive caregiving role to a more passive one [3]. Likewise, interactions between terminally ill parents and their children can be challenging. Research findings suggest parents’ struggle to tell their children about the serious medical condition in order to avoid distress and burden in their loved ones. Not telling, in turn, can cause emotional disturbances in their children [18]. So far, most investigators have examined burden and challenges in FMs and patients separately, but no study has focused on the interaction between patient and FMs, and, in particular, the relationship between parents and adult children. Examining the specifics of interaction between adult children suffering from a life-threatening disease at the EoL and their parents in comparison to the interaction of terminally ill parents and their children may provide a better understanding of the distinct psychosocial needs of both dyads.

## Study aims

The current research project seeks to explore the specifics of the interaction between a) adult children suffering from a life-threatening disease and their parents at the EoL in comparison to the interaction of b) terminally ill parents and their adult children. Both within-dyad as well as between-dyad interaction will be investigated. Detailed hypotheses regarding within-dyad differences are as follows: 1) Differences in the specifics of interaction in terms of communication, information about illness, expressed and perceived burden and support as well as caregiving role can be found between terminally ill children and their healthy parents. 2) Differences in the specifics of interaction can also be found between terminally ill parents and their healthy children. Pertaining to between-dyad differences, the authors want to explore the following hypothesis: 3) Differences with regard to the specifics of the interaction can be found between the two dyads (terminally ill children – healthy parents vs. terminally ill parents – healthy children).

Innovative aspects of this research project are, first, the topic under investigation which has not been systematically investigated to date. To the authors’ knowledge, no study exists which has explored the specifics of interaction in and between dyads of a) terminally ill children and their healthy parents, and b) terminally ill parents and their healthy children. Second, no evidence-based psychosocial intervention programs have been developed for both terminally ill adult children and their parents, as well as for terminally ill parents and their adult children. Third, the parallel use of quantitative and qualitative methods allows broadening the spectrum of information through the use of a multi-level and multi-perspective approach.

## Aims of study protocol

Research on interaction patterns of parents and their terminally ill adult children and adult children and their terminally ill parents raises a number of complex issues. These include field access, the challenge of recruiting parent-child dyads instead of independent individuals for study participation, the use of mixed methods, and the measurement of outcomes. The study protocol at hand is designed to shed light on these issues. Furthermore, the study protocol addresses ethical considerations, the subject of data security, and dissemination of study results.

## Methods and design

This study is a prospective observational study and aims at filling the gap on adult child-parent interaction specifics using an exploratory mixed-methods framework. The mixed-methods framework combines a qualitative semi-structured in-depth face-to-face interview and quantitative self-report questionnaires. Qualitative and quantitative data will be collected simultaneously in both dyads. Gysels et al. [19] have shown that qualitative interviews are suitable for PC patients and caregivers as they had therapeutic and empowering effects on participants.

The qualitative interview will focus on experiences, expectations, and wishes with regard to dyadic communication, information about illness and prognosis, expressed and perceived burden and support as well as caregiving role at the EoL. The questionnaires will cover socio-demographics, loneliness, attachment style, social support, and emotional closeness. Participants will provide information about their age, gender, citizenship, education, employment, relationship status, living arrangements, children, and primary caretaker responsibilities. Participants’ feelings of loneliness and social isolation are measured by the revised University of California, Los Angeles (UCLA) Loneliness Scale [20, 21]. The scale consists of 20 items for which participants rate how often they feel lonely or socially isolated on a Likert-type scale from 1 (never) to 4 (often). The Experiences in Close Relationships Scale (ECR) [22, 23] is a 36-item self-report measure that assesses individual differences with respect to attachment-related anxiety and attachment-related avoidance. Items are rated on a 7-point Likert scale from 1 (strongly disagree) to 7 (strongly agree). The 52-item Berlin Social Support Scales (BSSS) [24] measure six dimensions of cognitive and behavioral aspects of social support on a four-point Likert scale ranging from 1 (strongly disagree) to 4 (strongly agree). The Graphic closeness scale (GCS) [25] assesses individuals’ closeness on a one-dimensional line with oneself as anchor point. The scale ranges from 100 (very close) to 0 (not close = very distant).

A multiprofessional scientific advisory board (i.e. 9 experts from the fields of medicine, psychology, sociology, psychotherapy, medical ethics, hospice and PC work as well as bereavement, grief, and PC research) met in person on January 15, 2018 to provide valuable input for the research group on the development of the semi-structured interview guide, the selection of validated psychological questionnaires, and the refinement of recruitment strategy and cooperation practices. Furthermore, the board members made suggestions with regard to the socio-demographic questionnaire as well as questionnaire instructions in terms of clarity and methodological barriers.

### Study population and data collection

Study participants will be recruited via a German university medical center and further hospice and palliative care service providers. So far, three partners have agreed to aid in recruitment process. Further recruitment partners will be integrated during the course of the study if indicated. The ethics approval covers the integration of additional recruitment partners. Potential study eligibility will be assessed by the project managing study coordinator following the admission of a patient to the medical center’s PC unit, which holds seven beds, or to other cooperating wards or services offering E-o-L- care. Potential participants who meet the inclusion criteria (see section “Inclusion, exclusion, and termination criteria”) will be approached by a researcher. The researcher will invite both patient and FM/s to participate in a face-to-face interview and to fill in a set of questionnaires at the PC unit.

Due to the partly qualitative nature of this study, no precise sample size estimates or power calculations can be made at this point. Data collection will end once data saturation is obtained. Data saturation is achieved for the interviews when no more new or valuable information can be attained, further coding is no longer feasible, and once the study can be replicated [26, 27]. Existing exploratory qualitative studies in the area of grief and bereavement and palliative [12, 28–30] as well as qualitative research methods guidelines suggest that approximately 12 interviews are necessary to identify 90% of analysis codes [26]. The general patients’ and families’ willingness to participate in research is surprisingly high in palliative and E-o-L care [31]. Hence, inclusion of 12 to 20 terminally ill children and their parent/s as well as 12 to 20 terminally ill parents and their child/ren (a total of 24 to 40 patients and 24 to 40 FMs) for the qualitative interview and questionnaire battery is feasible. Hospice and PC cooperation partners will support study recruitment. So far, one hospital and one nursing service agreed for study cooperation. All study participants will be interviewed separately. If terminally ill children are survived by both parents and if terminally ill parents are survived by more than one adult child, all eligible FMs will be invited to participate in the face-to-face interview and to complete questionnaires during PC hospitalization or when receiving palliative or hospice care.

The collection of quantitative questionnaire data will be continued in both dyads even if saturation of qualitative interview data is reached. The assessment of a higher number of both described dyads enables better specification of the number of affected patients and FMs as well as testing of hypotheses and interrelations developed from qualitative data.

### Inclusion, exclusion, and termination criteria

Inclusion criteria for dyad *terminally ill children and healthy parents* comprise terminally ill patients 18 years of age or older who are seeking palliative or hospice care and who are in contact with at least one living parent, and for dyad *terminally ill parents and healthy children*, inclusion criteria comprise terminally ill parents who are seeking palliative or hospice care and who are in contact with at least one child age 18 years or older. Dyads of parents and children related through adoption or fosterage will be included. Having received comprehensive oral and written information about the nature, the content and aim of the study, and about study participation, both patient and FM/s provide written informed consent in order to be eligible to participate as a dyad in this project. Individuals of both sexes and all ethnic backgrounds independent of their main diagnosis may participate in the study when fulfilling eligibility criteria.

Patients and FMs will be excluded from study if they are cognitively impaired (e.g., patients with dementia), or if they are not sufficiently proficient in German.

Termination criteria include significant emotional distress during the interview and insufficient cognitive abilities to answer interview questions and/or questionnaires (a Mini-Mental-State Examination [32, 33] score < 24).

Inclusion, exclusion, and termination criteria apply to patients of both dyads and the respective dyadic partner (i.e., adult child/parent). In the case of the patient’s death prior to study participation, no interviews will be conducted with the relative. Already collected interview data will be discarded respectively. If FMs have not been interviewed at the time of the patient’s death but data exist for the deceased, FMs will be invited to participate after their loved one’s death. If they withdraw their study participation, the deceased’s data will be discarded.

### Data analysis

Starting point for dyadic data analysis is what might be termed the concept of “nonindependence” as outlined by Kenny et al. [34]: “Two members of a dyad are not simply two independent individuals. Rather, they share something in common that we refer to as *nonindependence*.” The mixed-methods design enables developing a comprehensive picture of the dyads: Questionnaire data will be used to describe participants and to distinguish potential sub-types within both dyads. For instance, one may explore whether dyads with high versus low scores in loneliness describe different communication barriers. This demarcation of dyad types will help draw conclusions regarding risks and challenges in E-o-L care for specific groups.

**Fig. 1:**
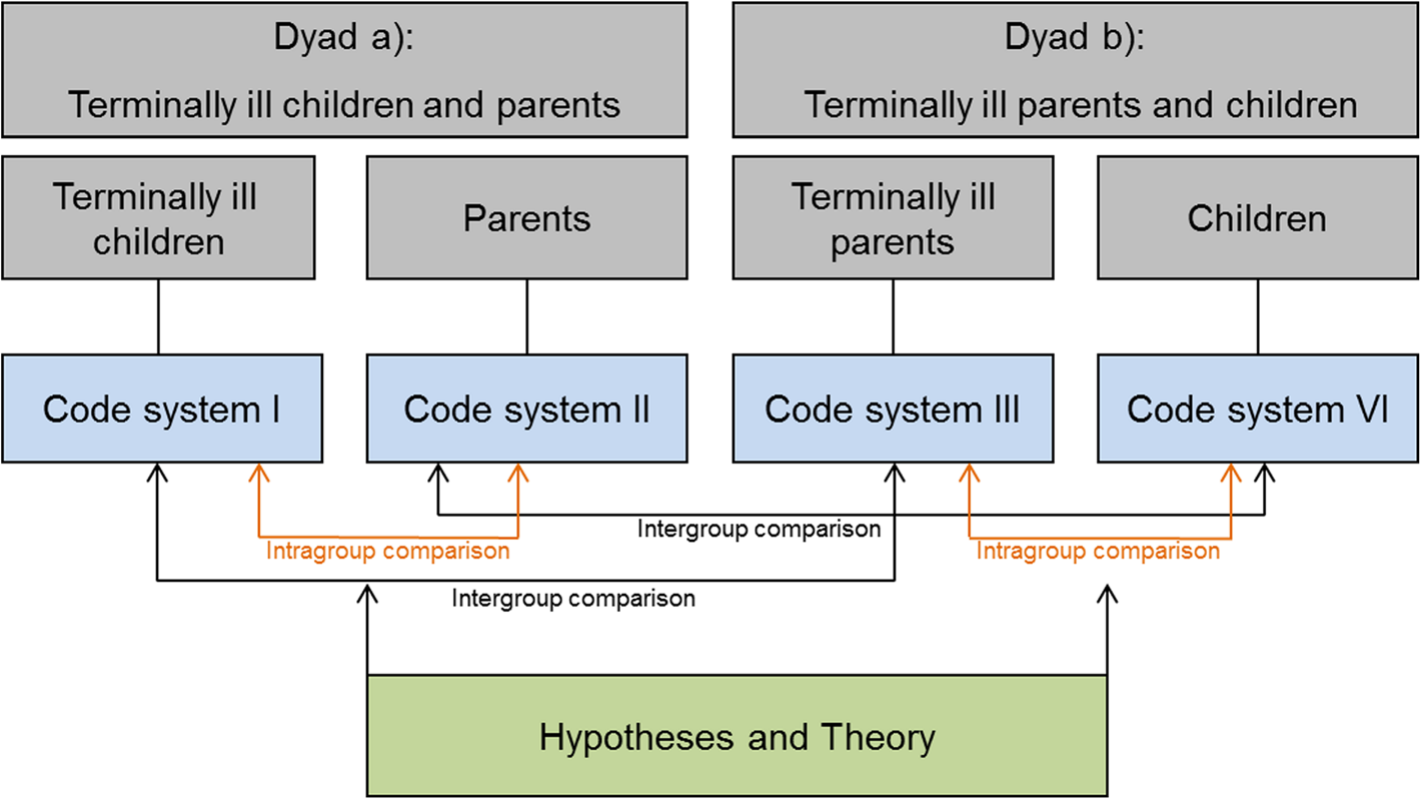
Development of code structure

Interviews will be transcribed verbatim by research assistants, coded by two independent researchers, and consented by a third researcher. All interviews will be analyzed qualitatively in MaxQDA [35] using methodological principles of Grounded Theory [36, 37]. This method is suitable to investigate social phenomena which are rather unknown so far. Grounded Theory allows a systematic understanding of peoples’ experiences and attitudes and is feasible even in vulnerable populations [38]. The bottom-up analysis technique allows to systematically code single text phrases (so-called “codes”) and to continuously summarize similar codes into more superior concepts. Finally, overall categories arise inductively. The current study will develop a code system for each unit of analysis which allows both intragroup comparisons within the dyads *terminally ill children and healthy parents* and *terminally ill parents and healthy children* and intergroup comparisons between the two dyads (see Fig. 1). From this structure, hypotheses can be built with regard to the addressed social phenomena. In a following step, a theoretical framework can be developed. This framework discloses main principles and connections between several aspects and assists in finding starting points for practical conclusions and ideas for interventions. Coding of interviews will take place in an iterative process in which interviews will be coded once conducted. The interview guide will be modified accordingly in the course of data collection if necessary.

The results of the “Grounded Theory” which are raised inductively from interview data will be augmented by results from standard questionnaires on loneliness, attachment, social support, and emotional closeness to enable a more detailed description of the study population. These well-established questionnaires will be applied to broaden the spectrum of information and to supplement the qualitative data, thus focusing on both depth and breadth of information [39], which is particularly beneficial for a small sample size. Quantitative data will be entered for statistical evaluation into the program IBM SPSS Statistics 24 (SPSS Inc., Chicago, IL, USA) for Windows and will then be analyzed according to the existing manuals. Prior to the main analysis, data cleaning and primary analysis will take place. Accuracy of data entries will be ensured through a series of steps [40], including evaluation of item range, checking for outliers, and calculation of univariate and bivariate statistics. Scale reliability will be assessed for all relevant variables. Descriptive statistics will be calculated to test assumptions of normality in the relevant outcome variables. Potential outliers will be examined. T-tests and chi-square tests will determine any group differences on demographic and outcome variables.

Finally, clinical conclusions on specific psychosocial care and support needs will be drawn. The members of the multiprofessional scientific advisory board will assist in data interpretation and development of recommendations for psychosocial support interventions in two face-to-face meetings. Recommendations regarding specific need-based psychosocial interventions will be drafted and consented (online) by board members. Volunteers (i.e. former caregivers and FMs whose relative/s received hospice and/or PC treatment) will serve as patient representatives and be included in a workshop. In addition, results will be presented in a patient and FM group for validation.

**Fig. 2:**
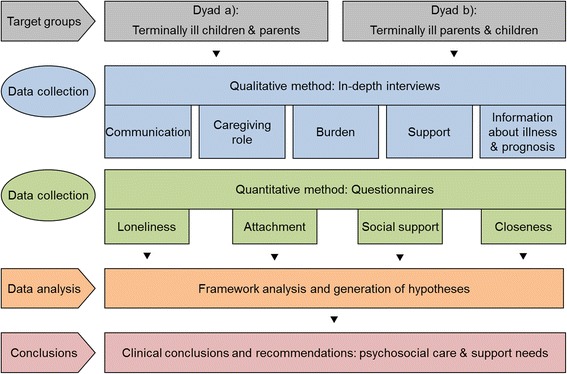
Study design

Figure 2 provides an overview of the study design. The figure illustrates the applied method of the development of research hypotheses and a theoretical framework on the specifics, similarities, and differences of both groups using Grounded Theory with its final aim of generating recommendations about specific psychosocial care needs.

### Expected results

The main expected results are i) a description of the specifics of the interaction within and between both dyads, ii) the development of hypotheses and a theoretical framework on the specifics, similarities, and differences for both study groups, and iii) clinical conclusions on specific psychosocial care needs of both groups. This knowledge will help infer the specific professional and semi-professional support needs of both patient-FM groups. The expected results will therefore improve PC practice.

## Discussion

The research group is currently incorporating the results of the multiprofessional scientific advisory board meeting (January 15, 2018). Semi-structured interview guide, sociodemographic questionnaire, questionnaire layout and instructions are being adjusted according to the board members’ suggestions. As a next step, and prior to qualitative and quantitative data collection, the interview questionnaires will be piloted at the palliative care unit of a German university medical center.

### Ethical considerations

Within this study, no interventions take place which may cause side effects. Prior to study participation, the study coordinator will explain the nature and purpose of the study, address methods of maintaining confidentiality, clarify the need to audio record the interview, and present and review the informed consent. Furthermore, participants will be asked if they understand the procedures to their full satisfaction and be encouraged to ask any questions they may have about the procedure. Remaining questions will be answered. The interview will take place once the participant has signed the consent form. Under no circumstances will coercion be applied to obtain informed consent. If participants wish to terminate the study at any time before completion or to withdraw from the study, they will be allowed to do so without naming any reasons.

If a participant experiences significantly high levels of discomfort or distress during the interview, the interview will be stopped and, if consented, continued later. All interviewers will receive interview training before study application to ensure empathetic and mild communication with vulnerable interviewees. Interviewers will be trained to observe specific signs of distress and burden and to provide crisis intervention. In addition, the multidisciplinary staff at the medical center will provide grief and support resources.

This is a low risk study. Due to the narrative nature of this study, participants may experience discomfort such as angry or sad feelings when talking about their or their loved one’s life-limiting illness/impending death. However, studies with comparable designs report low burden of participants [41, 42]. Additional resources will be provided to any individuals to counter discomfort or to participants who request them, and referral to counseling services will be suggested where indicated. The study coordinator and key researcher is experienced in working with and interviewing individuals in the E-o-L setting. She will be present or accessible during all interviews.

The research group can hardly predict whether participants will receive any benefits from study participation; however, scientific evidence suggests that individuals may benefit from study participation in the area of PC, grief, and bereavement [42–44]. Bereaved individuals describe study participation and talking about their loss as a helpful and positive experience even though studies may stir up negative emotions and can evoke painful memories. In addition, participants will contribute to an understanding of the needs of terminally ill individuals as well as of the factors that hamper and facilitate psychosocial adjustment. This knowledge, in turn, will help to improve future education and interventions. Study results will be shared with participants in the form of a letter once the results have been published in a peer reviewed journal.

The first interviews will be conducted by two interviewers in order to ensure consistency, whereas the following interviews will be performed by one interviewer only. Moreover, the interviewers will receive regular supervision.

### Data security

Confidentiality will be maintained through an identification number on all audio recordings and questionnaires. A document linking the participant’s name with their identification will be kept separately from the participant’s questionnaires. There will be no link between the participant’s identifying information and their identification number. Consent forms will be stored separately from data and kept in a locked filing cabinet. Digital files will be stored on password protected desktop computers and will be encrypted. Access to participant files will be limited to the study coordinator and her research team. When the results of the research are published or discussed at conferences, no information revealing participants’ identities will be included.

### Dissemination and implementation

Once data analysis is completed and written up, manuscripts will be published in peer-reviewed open-access journals. So far, no results of the study have been published. Moreover, results will be presented at national and international conferences (e.g. congresses of the European Association for Palliative Care and the German Association for Palliative Care) and disseminated via the section psychosocial care of the German Association for Palliative Care. Data files devoid of any personally identifiable information will be retained after study conclusion. In accordance with the American Psychological Association Ethical Principles of Psychologists and Code of Conduct, section 8.14 Sharing Research Data for Verification [45], the study coordinator will not withhold any pseudonymous data from other professionals who seek to verify the conclusions made by the author/s. If other professionals seek to answer new research questions using the data set, they need to gain permission from the research group and author/s.

The study project executing Institute for General Practice is well connected to the Medical Association of Lower Saxony (Ärztekammer Niedersachsen), the German Association for Palliative Care with their representation of Lower Saxony, the representation of Lower Saxony of the German Association of General Practitioners (Deutscher Hausärzteverband, Landesverband Niedersachsen e.V.), the Landesstützpunkt Hospizarbeit und Palliativversorgung Niedersachsen e.V., and the Palliativstützpunkt Hannover. These associations and support centers will be involved in public dissemination, transfer, and implementation of project results.

Educational workshops to communicate key results will be conducted for inpatient and community PC services. A report summarizing the main results will be provided on the website of the Institute for General Practice and be presented in relevant working groups as well as to practitioners. The project group aims at implementing and evaluating clinical recommendations for patient care in future projects. Results will further be presented at the annual scientific working days (Wissenschaftliche Arbeitstage) hosted by the German Association for Palliative Care. The working days are open to professionals as well as local organizations and the general public. So far, the study project and news from the first Scientific Advisory Board meeting have been publicized on the website of the executing Institute for General Practice [46, 47].

## Conclusion

This paper explains the purpose and function of the mixed-methods study as well as how to carry it out (i.e. the plan for conducting the mixed-methods study). Moreover, the present study protocol describes the method used to develop the semi-structured interview guide and explicates how questionnaires and the questionnaire instructions were tested in terms of clarity and methodological barriers. The authors aim at increasing transparency – particularly in the international context – by publishing the study protocol, thus making more information available than is currently being publicized in the two trial registries in which the project is indexed. The study protocol can be used by other researchers to prevent unnecessary duplication of the study project. Publication of the study protocol and later comparison with study results also give others the opportunity to see and understand deviations from the original study protocol that may occur during data collection and enables the scientific community to improve prospective study designs.

## Abbreviations

EoL/E-o-L: End of life/end-of-life
FM: Family member
PC: Palliative care
